# Proton therapy needs further technological development to fulfill the promise of becoming a superior treatment modality (compared to photon therapy)

**DOI:** 10.1002/acm2.13450

**Published:** 2021-11-03

**Authors:** Daniel E. Hyer, Xuanfeng Ding, Yi Rong

**Affiliations:** ^1^ Department of Radiation Oncology University of Iowa Iowa City Iowa USA; ^2^ Department of Radiation Oncology William Beaumont Hospital Royal Park Michigan USA; ^3^ Department of Radiation Oncology Mayo Clinic Arizona Phoenix Arizona USA

## 1 INTRODUCTION

There has been an ongoing debate over the superiority of proton beam therapy versus photon beam therapy for more than two decades.^1^, ^2^, ^3^ Proton versus photon has been just like two ends of a seesaw, one gaining superiority over the other as technologies advances during these years. Even though we are all familiar with the distinct benefit of the proton “Bragg Peak” for reducing exit dose compared to photon beams, there has never been a definitive answer as to which modality is superior. Additionally, Level I evidence demonstrating measurable clinical advantage of proton therapy is lacking. Superiority can be defined in two different aspects, the technology aspect and the clinical aspect. Technological superiority may or may not lead to superior clinical outcomes. From a physicist's point of view, it is safe to say that we would not see clinical outcome improvements if there is no clear advancement in the technology aspect. Yet in recent years, several major multicenter, prospective, randomized phase III trials comparing the two modalities have been initiated.^4^, ^5^ One might wonder, with the recent technology development in intensity‐modulated proton therapy based on pencil beam scanning, robustness optimization, etc., is proton therapy mature enough to enter such a level of clinical trials? In other words, do we have confidence that proton therapy is a superior treatment modality that should lead to positive improvement in clinical outcomes? Herein, Dr. Daniel Hyer argues for the proposition “*Proton therapy needs further technological development to fulfill the promise of becoming a superior treatment modality (compared to photon therapy)*,” while Dr. Xuanfeng Ding arguing against it.

Dr. Daniel Hyer received his PhD in Medical Physics from the University of Florida in 2010 and was certified by the American Board of Radiology in 2013 after completing his residency at the University of Iowa. Dr. Hyer is currently an Associate Professor and the Director of Clinical Physics at the University of Iowa. His research interests include MRI‐guided radiotherapy and proton beam therapy. On the latter topic, Dr. Hyer currently holds a National Cancer Institute grant as PI for the development of a proton collimator and has been actively engaged in proton therapy technological development for the past decade with two patents and over 30 peer‐reviewed manuscripts. He has also served as President of the Missouri River Valley chapter of the AAPM.

Dr. Xuanfeng Ding received his PhD in Physics from Wake Forest University in 2012, and finished his residency training at the University of Pennsylvania in 2014. After commissioning the first pencil beam scanning (PBS) compact proton system in Willis‐Knighton Cancer Center, Dr. Ding joined William Beaumont Hospital, Royal Oak, MI in 2015, as the lead proton physicist and Assistant Professor. Dr. Ding's research interests include proton arc technique, adaptive therapy, and motion management. He has received several extramural research grants as the PI and was granted multiple patents. Dr. Ding published over 30 peer‐reviewed papers and hundreds of conference abstracts. He is certified by the American Board of Radiology in Therapeutic Radiologic Physics. He also served as President of the Great Lakes Chapter AAPM and committee members of several AAPM Task Groups.

## 2 OPENING STATEMENT

### 2.1 Daniel E. Hyer, PhD

In its current form, proton therapy fulfills a niche role in radiation therapy. While it has been published that proton therapy *could potentially provide benefits* for approximately 15% of radiation therapy cases,^6^, ^7^, ^8^, ^9^ a much smaller percentage of patients are actually treated with proton therapy each year. Despite decades of clinical and technical research, why has proton therapy not emerged as a clinically dominant modality for the cases that might benefit? To answer this question, it is critical to understand the clinical advantage that proton therapy provides over photon therapy. Fundamentally, the advantage of proton therapy rests in the fact that protons have a finite range, virtually eliminating exit dose and significantly reducing the “low‐dose radiation bath” for a given treatment plan. This reduction in the low‐dose radiation bath is associated with a potential reduction in secondary malignancy rates in young patients^10^ and was the initial driving force to migrate proton therapy from the laboratory and into the clinical environment over the last 20 years. During this same time, photon therapy has continued to evolve and has seen numerous technological developments (intensity‐modulated radiotherapy [IMRT], volumetric modulated arc therapy [VMAT], image‐guided radiotherapy [IGRT], adaptive radiotherapy [ART]) that have successfully improved the conformity of the high‐dose radiation bath, and in some circumstances (IGRT and ART), allowed for the reduction in margins due to reduced setup uncertainties and the ability to account for daily anatomical variations. Entire treatment paradigms, such as stereotactic radiosurgery (SRS) and stereotactic body radiotherapy (SBRT), are now reliant on high‐dose conformity and small margins. While modern pencil beam scanning (PBS) proton therapy systems exhibit an improved conformity compared to initial double scattering systems, these systems still lag behind state of the art photon therapy systems in acheivable conformity for SRS and in some SBRT cases when accounting for range uncertainties.^11^, ^12^ These facts force clinicians using proton therapy to use larger margins and give up the ability to perform online adaptive therapy to capture the potential benefits of improved conformity of the low‐dose radiation bath. In some cases, this tradeoff may be reasonable, especially in young patients with large clinical target volumes. However, I argue that if proton therapy is going to reach its full potential as a superior treatment modality, further technological developments are needed to eliminate such tradeoffs.

First and foremost, proton therapy needs better online imaging systems. This has been especially evident in recent years as photon therapy has blossomed with onboard fan‐beam CT (FBCT) and MR imaging systems, while proton therapy has only recently been equipped with commercially available gantry mounted cone‐beam CT (CBCT). The lack of integrated high‐contrast and high‐resolution imaging puts proton therapy at an inherent disadvantage with respect to planning target volume margins required to account for setup uncertainties. This is exacerbated by the fact that proton therapy is also more sensitive to patient misalignments and anatomical changes due to the finite particle range and associated steep distal dose fall‐off. Investigators have proposed MR guidance for adaptive proton therapy to address this challenge,^13^, ^14^ and it is reasonable to believe a technological development of this magnitude will be necessary to allow clinicians to minimize uncertainties associated with daily anatomical changes using a proton therapy approach.^15^

As outlined in the introduction, improvements in the high‐dose conformity are also necessary for some cases.^11^, ^12^, ^16^ Since protons are often used to treat sensitive areas in the brain or in pediatric patients, improving the high‐dose conformity is critical to achieve favorable long‐term outcomes. It has previously been shown that the lateral dose conformity in PBS proton therapy can be improved by reducing the beam size, but this is a difficult task to achieve, especially when treating superficial targets which requires the use of low‐energy beams.^12^, ^16^ One potential method to reduce the effective beam size is through the use of advanced collimation systems designed specifically for proton therapy.^17^, ^18^, ^19^, ^20^, ^21^ In preclinical studies, utilization of collimation systems have been shown to provide a substantial improvement in high‐dose conformity when treating both brain and head and neck cancers.^22^, ^23^, ^24^ These dosimetric advantages are far superior to traditional aperture‐based collimation approaches^25^, ^26^, ^27^ while still yielding robust treatment plans.^28^ Arc geometries have also recently been proposed as an alternative method to improve high‐dose conformity of proton therapy.^29^, ^30^ In addition to conformity improvements, by spreading out the treatment over an entire arc instead of just a few treatment angles, range uncertainty is distributed over a greater volume rather than just a few limited directions, thereby improving plan robustness.^31^

The final development that warrants discussion is the accurate determination of relative biological effectiveness (RBE). Currently, the RBE for clinical proton therapy is assumed to be 1.1, regardless of beam energy or tissue type. However, based on the characteristics of the Bragg peak and the increased linear energy transfer at the end of the proton track, it is universally known that the true RBE is not a constant. In fact, protons have been shown to reach an RBE of up to 3 in the Bragg peak via in vitro studies.^32^, ^33^ Unfortunately, RBE depends on complex physical and biological responses and is difficult to accurately calculate. Once these biological effects are better understood, treatment planning system developments that simultaneously optimize placement of particle linear energy transfer (LET) along with RBE dose may hold the key to unlocking the full dosimetric advantage of proton therapy.^34^ In the meantime, our limited understanding of proton RBE limits our options from a proton treatment planning perspective. With current planning systems, it is difficult to ascertain where high‐LET particles are expected in the patient, which in turn limits our ability to assess plan quality and response.

In summary, without technological advances in online imaging, collimation, arc delivery, and RBE‐based treatment planning, proton therapy will fall short of achieving its full clinical potential.

### 2.2 Xuanfeng Ding, PhD

In this debate, I will explain why proton therapy has already fulfilled its promise as a superior treatment modality at its current technology level from a physicist's point of view. First, proton therapy has demonstrated a substantial advantage over photon therapy by utilizing its unique characteristic, “Bragg Peak.” More specifically, the integral dose with proton is about 60% lower than the photon technique.^35^ While it appears most cost‐effective for pediatric patients maximizing the clinical gain, it is also essential for adult patients for minimizing the dose to any healthy tissue.^36^ In fact, the motivation to further reduce dose to patient's healthy organs has been a driving factor of technology innovations in the radiation oncology community including higher dose conformality, improved on‐board volumetric imaging, motion management devices, etc.^37^, ^38^, ^39^, ^40^ Thus, the capability of integral dose reduction itself is evidence of a superior treatment modality. Such advantage offers a safe and effective curative reirradiation strategy for varieties of disease sites including recurrence of head–neck cancer, chordoma, non‐small cell lung cancer (NSCLC), and breast cancer.^41^, ^42^, ^43^, ^44^

Additionally, the IMPT, based on the PBS technique,^45^ has further improved the normal tissue dose sparing both in the distal end and the proximal end of the target volume. In the last decade, the vast majority of the new proton institutions worldwide have adopted IMPT in routine clinical practice.^46^ Numerous peer‐reviewed publications have shown its significant dosimetric advantages over a wide range of clinical indications compared to photon therapy, not only in the integral dose reduction but also in the improvement of medium‐ and high‐dose conformality.^47^, ^48^, ^49^, ^50^, ^51^, ^52^, ^53^, ^54^, ^55^ More interestingly, the results from ProKnow (Elekta, Stockholm, Sweden) planning competitions showed IMPT plans dominated the top plan quality scores in the recent challenging cases such as GYN(2018), Liver SBRT(2020), and advanced‐stage lung cancer(2021). Though these planning exercises depend on the planners’ skills, availability, and experience, it provides strong support to the IMPT 's superiority over photon technique.^56^

Furthermore, such dosimetric advantage increases the tolerance of chemotherapeutic agents by reducing toxicity and permitting higher drug doses than photon therapy. A phase II study of concurrent chemotherapy for unresectable stage III NSCLC showed promising clinical outcomes and rate of toxic effect compared with historical photon therapy data.^57^ When studying the structural and hemodynamic changes of contralateral healthy brain tissue following proton and photon radiochemotherapy, Petr et al. reported a reduced brain‐volume loss in the proton therapy group.^58^ A more recent analysis of 1483 adult patients with nonmetastatic, locally advanced cancer treated with concurrent chemoradiotherapy showed that proton chemoradiotherapy was associated with significantly less acute adverse events, for example, 90‐day unplanned hospitalizations.^59^ These emerging clinical evidence filled the immediate needs of toxicity and side‐effect mitigation utilizing concurrent chemo‐RT that photon therapy cannot offer.

Dose‐escalation strategies are generally considered to offer better local tumor control. However, it was sometimes associated with more side‐effects in some challenging disease sites due to the dosimetric limitation of photon therapy in which more radiation dose spills into the healthy tissue. For example, a phase III study in prostate cancer treatment showed increased acute bowel and bladder reaction as well as late rectal side‐effects.^60^ RTOG 0126, a randomized clinical trial for intermediate‐risk prostate cancer (1532 patients), concluded that photon therapy dose escalation did not improve overall survival, and high dose resulted in more late toxic effects.^61^ The result from a randomized clinical trial for inoperable stage III NSCLC, RTOG 0617, concluded that the photon dose escalation provides no benefit in overall survival, and it might be potentially harmful.^62^ Therefore, more effective and safe treatment techniques are urgently needed for these cancer patients. In contrast to the photon randomized clinical trial results, Gomez et al. summarized the proton clinical experience from multiple institutions. They found that hypofractionated dose‐escalated proton therapy for NSCLC is feasible, and the evidence is more substantial for early‐stage disease.^63^ Additionally, Chan et al. reported their preliminary finding that dose escalation with proton resulted in low toxicity and high local control rate in the treatment of high‐grade meningiomas.^64^ With the superiority of the dosimetric distribution, the role of proton therapy in dose‐escalation strategies has been gradually becoming evident.

Most importantly, proton therapy has entered its golden stages of technological development. Numerous novel concepts and treatment delivery technologies are under investigation such as dynamic collimator system^21^ and spot‐scanning arc therapy.^29^ The future of proton therapy has been brighter than any other time in the era of modern radiotherapy. On the other hand, the development of photon treatment delivery technique has reached its ceiling.

In summary, despite the lack of level 1 clinical evidence, the existing proton therapy technology has demonstrated its superiority in varieties of aspects: (1) dosimetric characteristics in a broad range of disease sites, for example (1a) less integral dose; (1b) better plan quality; (2) immediate clinical needs, for example, (2a) reirradiation; (2b) concurrent chemo‐RT; (2c) dose escalation. Such advantages will be more prominent in the near future because of its greater potential and higher ceiling compared to the photon therapy.^65^

## 3 REBUTTAL

### 3.1 Daniel E. Hyer, PhD

I will start my rebuttal by stating that I agree with Dr. Ding—the future of proton therapy is exceptionally bright and there are many promising avenues of research that could substantially increase the relevance of this modality. However, in its current state, it is hard to argue against the need for further technological development of proton therapy. The main evidence that Dr. Ding uses to demonstrate the current superiority of proton therapy includes integral dose reduction, treatment planning studies/competitions, and a variety of dose‐escalation clinical trials, none of which represent a high level of evidence.

The issue with treatment planning studies is that they assume a single anatomical image and reference anatomy, usually concluding that a specific dosimetric criterion is superior in one case over another. Such theoretical studies may be reasonable when comparing intramodality (i.e., photons versus photons), but are largely inadequate to provide direct intermodality comparisons in practice. For example, we know that proton therapy is much more sensitive than photon therapy to pathlength differences and range uncertainties. A fair comparison between photons and protons must consider the robustness of the plans to daily anatomical variations and intrafraction motion, an area where photon‐based modalities hold a key advantage due to the lack of a finite range. Ultimately, adaptive proton therapy is an example of a technological advancement required to overcome this challenge. Such a development would ensure that the radiation dose is placed appropriately with respect to daily anatomy.

With regards to dose escalation, the comparisons given by Dr. Ding reach across multiple disease sites, stages, and modalities. It is difficult to conclude a direct correlation between protons and photons from these studies. However, if these studies are indeed applicable in this type of comparison, they demonstrate that at a minimum we need more knowledge regarding the biological effects of proton therapy. Without such knowledge, we may see unintended consequences, such as brainstem toxicities, that have been observed in pediatric patients receiving PBS proton therapy.^66^ Further development surrounding the biological effects of proton therapy would not only help us avoid such negative side‐effects, but also begin to reveal why proton therapy may be beneficial in some cases and the scenarios where its application pays the greatest dividends. Currently, our best guess is that a reduction in integral dose is responsible for the improvements in outcomes for some cases, but I believe integral dose only tells part of the story.

Lastly, Dr. Ding states that integral dose with proton therapy is less than that of contemporary photon techniques. I agree with this statement, but at the same time must conclude that a reduction in integral dose alone is not enough to unequivocally demonstrate proton therapy as a superior modality for most cases. There are certainly promising studies that make a compelling case that the reduction in the low‐dose radiation bath could meaningfully improve secondary malignancy rates in very young patients, xerostomia in head and neck cancer patients, and cardiac injury from lung cancer treatment.^10^, ^54^, ^67^ I argue that these investigations demonstrate the importance and ongoing need to technically develop and clinically study the promising field of proton therapy as a community. They exemplify the potential of proton therapy, but more work is needed for proton therapy to fulfill its promise of becoming a superior treatment modality.

### 3.2 Xuanfeng Ding, PhD

Dr. Hyer has raised an excellent question: “*Why has proton therapy not emerged as a clinically dominant modality for the cases that might benefit?*” In my opinion, it is not because of the limitations in the dosimetric plan quality or technology. Instead, the investment cost and accessibility to our communities are the two major factors limiting our patients' chance to choose such a superior treatment modality. The initial investment of a proton therapy center could cost from $20 million to $200 million on top of the high operation and maintenance cost.^68^ As a result, there are only about 90 particle therapy centers worldwide, where 40 of them are located in the US compared to approximately 1500 cancer centers nationally.^69^, ^70^ Every patient deserves a better treatment option. Unfortunately, even with 24‐h shifts in all these PTCs, there is no way to meet such clinical demands. Sometimes it requires a justification of cost‐effectiveness utilizing such a precious medical resource.^71^, ^72^ Though the scale of financial benefit to the patient population, the healthcare system, and society may vary among different countries and policies,^73^, ^74^, ^75^, ^76^, ^77^ one thing was found in common that there are potential benefits in terms of cost and quality‐adjusted life‐years for many disease sites.^72^, ^76^ Excluding the economic factors, the normal tissue complication probability model‐based patient selection system for head and neck cancer was implemented in the Netherlands as a National Indication Protocol.^78^ Such a protocol served as an excellent starting point and an example of serving more cancer patients in need of advanced treatment modalities worldwide. We will see more countries and investigations to join such direction for varieties of clinical indications.^79^, ^80^

This debate is not on “clinically dominant modality” but on the technological superiority comparison. The imaging system has been a critical part of radiation therapy. I agree with Dr. Hyer that integrating new imaging techniques in a proton system is relatively slower than photon radiotherapy due to the cost and engineering challenges. FBCT is always nice to have. Today, CT on‐rail has been clinically implemented in some proton institutions, which provides valuable information for adaptive therapy.^81^ The current CBCT on proton gantry is also sufficient for adaptive planning decisions utilizing the artificial intelligence‐based synthetic CBCT approach. Such a platform allows a direct proton dose calculation on the daily CBCT,^82^ which has been clinically implemented in some institutions to assist the adaptive planning decision including Beaumont proton therapy center.^83^ MR‐guided proton system is an exciting topic that is now under active research and development and is expected to be clinically available in 5–10 years.^13^ This technique will provide additional benefits to proton therapy.

RBE uncertainty is indeed one of the major challenges. However, the emerging evidence showed that LET is correlated to the clinical endpoint, for example, rectal bleeding^84^ and MRI changes in the normal brain tissue.^85^ With the current IMPT technique, we could optimize such a physical parameter accurately and directly during the planning without worrying too much about the RBE uncertainties.^86^

In summary, current technologies and tools in proton therapy have sufficiently addressed the outstanding issues such as adaptive proton therapy based on CBCT and CT on‐rail, robust optimization/evaluation,^87^ and LET optimization/evaluation. The clinical evidence is growing in favor of proton beam therapy translated from its dosimetric advantage and less integral dose. Admittedly, there are still opportunities to further develop the proton therapy technique by sharping the dose fall‐off and integrating MR‐guided system, but these incremental improvements will not affect the overall picture of its superiority in the dosimetric advantage and clinical benefits as of today.

## AUTHOR CONTRIBUTION

DH and XD contributed in drafting the manuscript, YR contributed in moderating and modifying the paper.
